# Hip arthroscopy following contralateral total hip arthroplasty: a multicenter matched-pair study

**DOI:** 10.1093/jhps/hny047

**Published:** 2018-12-07

**Authors:** Karan A Patel, Benjamin G Domb, Aaron J Krych, John M Redmond, Bruce A Levy, David E Hartigan

**Affiliations:** 1 Department of Orthopedics, 5777 East Mayo Blvd, Phoenix, AZ, USA; 2 Department of Orthopedics, American Hip institute, 1010 Execturive Court Suite 250 Westmont, IL, USA; 3 Department of Orthopedics, 200 First St SW, Rochester, MN, USA; 4 Department of Orthopedics, 2627 Riverside Ave, Suite 300 Jacksonville, FL, USA

## Abstract

The purpose of this study was to determine if patients undergoing hip arthroscopy for labral pathology with contralateral total hip arthroplasty (THA) have a difference in revision surgeries or patient-reported outcomes (PROs) when compared with those patients undergoing hip arthroscopy for labral pathology with a native contralateral hip. A retrospective review was performed for patients that were undergoing hip arthroscopy between 2008 and 2015. Patients were included in the study group if they met the following inclusion criteria: Tönnis Grade 0 or 1, hip labral pathology, previous contralateral THA, and greater than 2-year follow-up with completion of all PROs or conversion to a THA. Exclusion criteria included the previous surgical history on ipsilateral hip, peritrochanteric or deep gluteal space arthroscopy performed concomitantly, or dysplasia [Lateral Center Edge Angle (LCEA) < 20°]. A 3:1 matched-pair study was conducted. Multiple PRO scores were recorded for both groups. There was no statistically significant difference in the modified Harris hip score, non-arthritic hip score, hip outcome score-sports specific sub-scale, visual analog pain score and patient satisfaction scores between both groups. However, the study group was noted to have six patients converted to THA (67%) at an average of 30 months post-operatively, compared with only four patients (15%) in the control group (*P* = 0.006). Hip arthroscopy cannot be currently recommended in patients who have undergone contralateral THA due to the high conversion to THA (67%).

## INTRODUCTION

Total hip arthroplasty (THA) is one of the most common and successful orthopedic procedures performed worldwide. The rate of THA is predicted to increase 174% by 2030 [1]. Indications for THA are variables; however, over 90% of patients who undergo THA have a primary diagnosis of osteoarthritis [2]. Over 85% of patients report being completely satisfied following THA, making it one of the most successful orthopedic surgeries performed [3]. A clinically significant improvement in patient-reported outcomes (PROs) and quality of life have also been reported [4–12].

The improvement in technology and technique of hip arthroscopy has enabled treatment of pathology at an earlier age, in hopes of preventing the continued degeneration of the joint due to offending osseous and soft tissue pathoanatomy. Hip arthroscopy continues to gain popularity as there is strong evidence for patient improvement following arthroscopy [13–23]. As indications for hip arthroscopy continue to expand, an increasing number of aging patients meet criteria for this intervention and are not yet candidates for THA. One of the most common indications for hip arthroscopy is a labral tear with femoroacetabular impingement. Previous studies have noted a conversion to THA following hip arthroscopy between 4 and 25% [19, 24, 25]. Risk factors for failure of hip arthroscopy include obesity, decreased joint space (<2 mm), significant chondromalacia, Tönnis Grade 2 osteoarthritis and remaining unaddressed FAI [24, 26].

Patients that undergo hip arthroplasty have demonstrated a 15% chance of arthroplasty and 30% chance of development of radiographic osteoarthritis on the contralateral hip at 10 years [27]. At this point, it is uncertain if intervention in symptomatic patients can improve these results by treating labral pathology and bony dysmorphisms prior to the onset of arthritis.

Patients that have undergone hip arthroplasty and have significant hip pain unresponsive to conservative treatment in the contralateral hip, while having intact joint space and the pathoanatomy of femoroacetabular impingement, are a difficult patient population to treat. The patient’s drastic improvement following their THA may set unrealistic patient expectations for any procedure that is not able to provide similar results. This study seeks to answer the following questions: (i) Is the conversion to THA higher in patients who have previously had a contralateral THA? (ii) Do patients undergoing hip arthroscopy for labral tears following contralateral THA have a clinically meaningful improvement in their PROs? (iii) Are PROs significantly different in patients undergoing hip arthroscopy following contralateral THA compared with those who have not had a contralateral THA?

The authors hypothesize patients undergoing hip arthroscopy following contralateral THA will (i) Have higher conversion rates to THA compared to patients who have not gone through a contralateral THA. (ii) Have a clinical meaningful improvement in PROs. (iii) Have similar improvement in PROs compared with those patients who have not gone through a contralateral THA.

## MATERIALS AND METHODS

This research study was reviewed and approved by Mayo and American Hip Institute Institutional Review Board (#16-009534).

Both sites prospectively collected data on hip arthroscopy patients and these data were retrospectively reviewed for patients that were undergoing hip arthroscopy between 2008 and 2015. Patients were included into the study group if they met the following inclusion criteria: Tönnis Grade 0 or 1, hip labral pathology, previous contralateral THA and greater than 2-year of follow-up with completion of all PROs or conversion to a THA. Exclusion criteria included previous surgical history on ipsilateral hip, peritrochanteric or deep gluteal space arthroscopy performed concomitantly or dysplasia [Lateral Center Edge Angle (LCEA) < 20°]. Control group was collected by matching the following criteria: age at surgery ±5 years, gender, BMI ± 5, labral treatment, acetabuloplasty (yes or no), femoroplasty (yes or no), pre-operative Tönnis grade, outerbridge classification (femoral head and acetabulum) and pre-operative LCEA ± 5° (Tables I and II).

**Table I.** Patient demographics in both groups demonstrating no significant differences

	Control (*n* = 27)	Contralateral THA (*n* = 9)	P-value
**Patients and hips included in study**			
Left	12	2	0.432
Right	15	7	
**Gender**			
Male	15	5	1.000
Female	12	4	
**Age at surgery (years, mean, SD, range)**	48.2±6.7 (4.0–63.3)	49.2±7.3 (40.5–63)	0.730
**BMI (mean, SD, range)**	28.5±4.8 (20.3–40.3)	28.4±5.6 (22.5–40.6)	0.952
**Tonnis Osteoarthritis Grade**			
Grade 0	24	8	1.000
Grade 1	3	1	
**LCEA (degrees, mean, SD, range)**	27.7±6.2 (15–40)	27.4±7.1 (19–42)	0.917

**Table II.** Intraoperative findings in both groups, demonstrating no significant differences

	Control (*n* = 27)	Contralateral THA (*n *= 9)	P-value
**Seldes-type tear**			
1	10 (37.0%)	3 (33.3%)	1.000
2	7 (25.9%)	1 (11.1%)	0.648
1 and 2	10 (37.0%)	5 (55.6%)	0.443
ALAD			
0	3 (11.1%)	4 (44.4%)	0.050
1	2 (7.4%)	1 (11.1%)	1.000
2	10 (37.0%)	0 (0%)	0.039
3	8 (29.6%)	0 (0%)	0.160
4	4 (14.8%)	4 (44.4%)	0.086
**Outerbridge (acetabular)**			
0	3 (11.1%)	3 (33.3%)	0.151
1	1 (3.7%)	1 (11.1%)	0.443
2	12 (44.4%)	1 (11.1%)	0.114
3	4 (14.8%)	0 (0%)	0.553
4	7 (25.9%)	4 (44.4%)	0.409
**Outerbridge (femoral head)**			
0	18 (66.7%)	3 (33.3%)	0.122
1	1 (3.7%)	0 (0%)	1.000
2	2 (7.4%)	3 (33.3%)	0.088
3	5 (18.5%)	2 (22.2%)	1.000
4	1 (3.7%)	1 (11.1%)	0.443
**LT percentile class (Domb)**			
0–0%	11 (40.7%)	3 (33.3%)	1.000
1–0%<50%	11 (40.7%)	3 (33.3%)	1.000
2–50%<100%	5 (18.5%)	3 (33.3%)	0.384
3–100%	0 (0%)	0 (0%)	1.000
**LT villar class**			
0—no tear	11 (40.7%)	3 (33.3%)	1.000
1—complete rupture	0 (0%)	0 (0%)	1.000
2—partial tear	7 (25.9%)	2 (22.2%)	1.000
3—degenderate tear	9 (33.3%)	4 (44.4%)	0.693

Along with patient demographics, the following was retrieved from the chart review: procedure performed, intraoperative findings [labral tear type, Outerbridge score [28] and Ligamentum Teres status (Villar class, Domb class)] [29], secondary operation (revision arthroscopy, THA), PRO, visual analog pain score (VAS) and patient satisfaction in both the control and study group (scale of 1–10, with 10 being completely satisfied). PROs were administered pre-operatively as well as post-operatively at 6 months, 1 and 2 years. The PROs obtained were the modified Harris hip score (mHHS), non-arthritic hip score (NAHS) and hip outcome score-sports specific sub-scale (HOS-SSS). Patients in the study group had PRO scores collected at all time points unless a conversion to THA occurred. They were considered failures at this time point and no PRO scores were reported. These PROs have been shown to have high clinometric properties [30–33].

Radiographs were taken pre-operatively, at the first post-operative visit and annually thereafter. Each patient had an AP of the pelvis, false profile view and 45° Dunn view. These radiographs were utilized to measure the LCEA, anterior center edge angle, alpha angle, Tönnis angle and Tönnis grade of osteoarthritis. Prior to arthroscopy, all patients had undergone a dedicated hip MRI scan to assess the patient’s cartilage as well as the patient’s labrum.

### Surgical technique

All hip arthroscopies were performed by one of the three physicians with greater than 5 years of experience in hip arthroscopy (B.G.D., A.J.K. and B.A.L). All procedures were performed at high volume hip arthroscopy centers. Surgeries were performed in the supine position utilizing a minimum of two portals (standard anterolateral and mid-anterior), and when labral repair was conducted a distal lateral accessory portal was also created. After establishment of portals and inter-portal capsulotomy, a diagnostic arthroscopy was carried out. Cartilage at the acetabular labral interface was documented utilizing the acetabular labral articular disruptions (ALADs) [34]. Cartilage damage on the acetabular and femoral side was calculated utilizing Outerbridge classification [28]. Bony cam and pincer resection were corrected under fluoroscopic and arthroscopic guidance. Labral tears were repaired if possible and if not then the labrum was selectively debrided until a stable labrum was achieved, while preserving as much labral tissue as possible to maintain the labrums suction seal. If there was full-thickness cartilage damage present, the size and location of the lesion were noted using a 5-mm probe and the clock-face method [35].

### Rehabilitation

All patients were foot flat weight-bearing (20 lbs or less) for 2 weeks post-operatively. Thereafter, they were gradually allowed to return to weight-bearing as tolerated. All patients started physical therapy on the first post-operative day to initiate range of motion. This was accomplished by using a continuous passive motion machine for 4 h per day or using a stationary bicycle for 2 h per day. All patients were placed on 500 mg of Naprosyn bid for 6 weeks for inflammation and heterotopic ossification prophylaxis.

### Statistical analysis

Based on an a priori power analysis, it was estimated that a clinically significant difference between both groups in regards to mHHS would be 8.0, with a standard deviation of the pre-operative group being 10. After performing a Cohen’s *d* calculation to compute the two-tailed effect size for a *t* test for independent samples, it was determined that a 1:3 group-matching ratio must be achieved to attain a power of 0.8 or higher. Study group sample size was calculated to be 9. Statistical analysis was performed using Microsoft Excel 2007 (Microsoft Corp). Shapiro–Wilk test was used to evaluate the normality of the data. Student *t* test was used to compare normally distributed data and a Wilcoxon Signed-rank test was used for non-normally distributed data. A *P*-value <0.05 was considered statistically significant.

## RESULTS

A total of 12 patients of 2089 hip arthroscopies performed in both centers (0.57%) were identified, and 9 met inclusion criteria and were included in this study as they had a 2-year minimum follow-up. The patients who remained in the study group (i.e. did not have a THA) had an average follow-up of 30 months (SD 2.6). There were 2089 patients eligible in the same period for the matched cohort. A 3:1 matched-cohort group was established using the parameters discussed in the methods section. The control group had an average follow-up of 46.3 months (SD 22.6). There was no statistical difference between the study group and control group for laterality, gender, age, BMI, follow-up time, Tonnis grade or LCEA (Table I). There was also no statistical difference on intraoperative findings including Outerbridge classification (femoral head and acetabulum), ALAD, labral tear type and treatment type (Tables II and III).

**Table III.** Procedures performed in both groups, demonstrating no significant differences

	Control (*n* = 27)	Contralateral THA (*n* = 9)	P-value
**Labral treatment**	* *	* *	* *
Repair	9 (33.3%)	3 (33.3%)	1.000
Debridement	18 (66.7%)	6 (66.7%)	1.000
**Acetabuloplasty**	15 (55.6%)	5 (55.6%)	1.000
**Femoroplasty**	27 (100%)	9 (100%)	1.000
**Capsular treatment**			
Repair/plication	10 (37.0%)	3 (33.3%)	1.000
Release	17 (63.0%)	6 (66.7%)	1.000
**Notchplasty**	7 (25.9%)	4 (44.4%)	0.409
**Ligamentum teres debridement**	7 (25.9%)	2 (22.2%)	1.000
**Removal of loose body**	5 (18.5%)	4 (44.4%)	0.184
**Iliopsoas fractional lengthening**	6 (22.2%)	1 (11.1%)	0.652
**Synovectomy**	6 (22.2%)	1 (11.1%)	0.652
**Acetabular microfracture**	5 (18.5%)	3 (33.3%)	0.384
**Femoral head microfracture**	0 (0%)	0 (0%)	1.000
**Acetabular chondroplasty**	1 (3.7%)	1 (11.1%)	0.443
**Femoral head chondroplasty**	1 (3.7%)	1 (11.1%)	0.443
**Trochanteric bursectomy**	7 (25.9%)	2 (22.2%)	1.000
**Gluteus medius/minimus repair**	3 (11.1%)	2 (22.2%)	0.581

PROs are documented in Table IV. Of note, the patients in the study group who converted THA did not have PROs included in the final analysis, as THA was treated as the end point. There was no statistically significant difference in the mHHS, NAHS, HOS-SSS, VAS and patient satisfaction scores between both groups. There was no statistical difference between pre-operative and post-operative PRO scores for the study group; however, the absolute difference was similar to the improvement seen in the control group.

**Table IV.** *Patient-reported outcomes between the two groups, as well as each group pre-operatively* and at last follow-up

	Control (*n* = 8)	Contralateral THA (*n* = 3)	*P*-value
**Follow-up time (mean, SD)**	46.3±(22.6	30.0±(2.6	0.399
**mHHS (mean, SD)**			
Pre	57.1±(15.9	53.3±(10.3	0.714
Latest	82.2±(21.3	92.3±(10.8	0.609
*Δ*	25.1±(20.3	39.0±(19.0	0.33
Pre–post *P*-value	**0.01**	0.071	
**NAHS (mean, SD)**			
Pre	57.3±(21.7	56.3±(2.4	0.945
Latest	81.6±(21.7	75.8±(21.5	0.975
*Δ*	24.3±(17.6	26.6±(22.3	0.862
Pre–post *P*-value	**0.005**	0.176	
**HOS-SSS (mean, SD)**			
Pre	38.2±(25.9	34.4±(25.6	0.855
Latest	74.5±(31.7	94.4±(3.9	0.420
*Δ*	32.7±(32.3	60.1±(18.2	0.302
Pre–post *P*-value	**0.0369**	a	
**VAS (mean, SD)**			
Pre	6.23±(2.2	7.62±(0.5	0.310
Latest	2.57±(2.9	2.24±(1.9	0.310
*Δ*	−3.6±(3.3	−5.4±(1.6	0.414
Pre–post *P*-value	**0.011**	**0.027**	
**Patient satisfaction (mean, SD)**	8.3±(2.3	6.3±(4.0	0.475

^a^Only two patients in CL THA group.

**Fig. 1.**
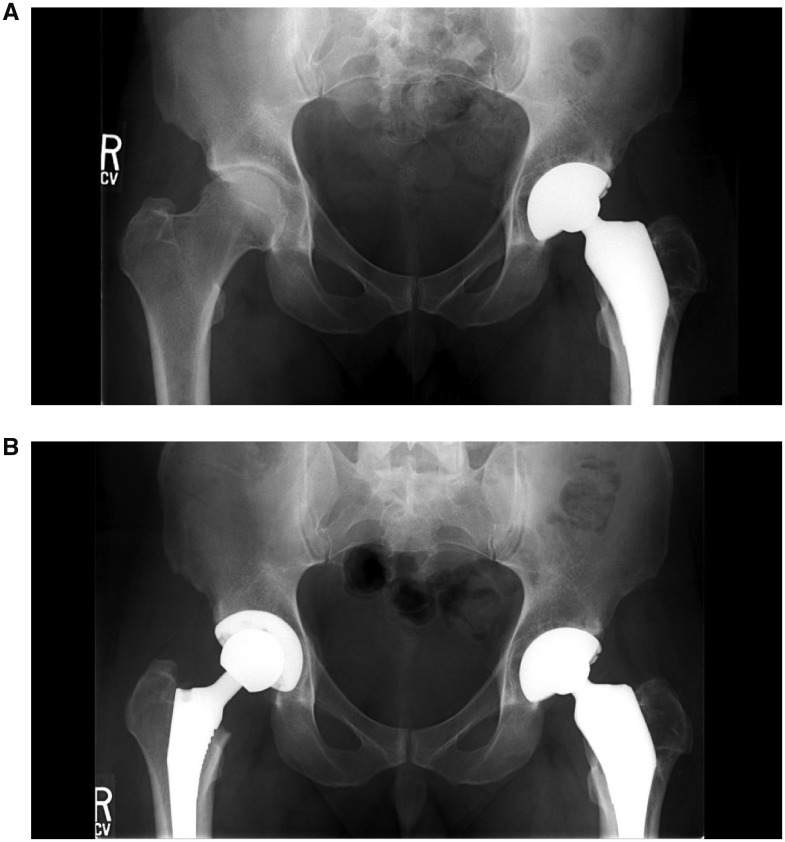
Pre-operative (**A**) and post-operative (**B**) radiographs for patients undergoing THA in the study group.

**Fig. 2.**
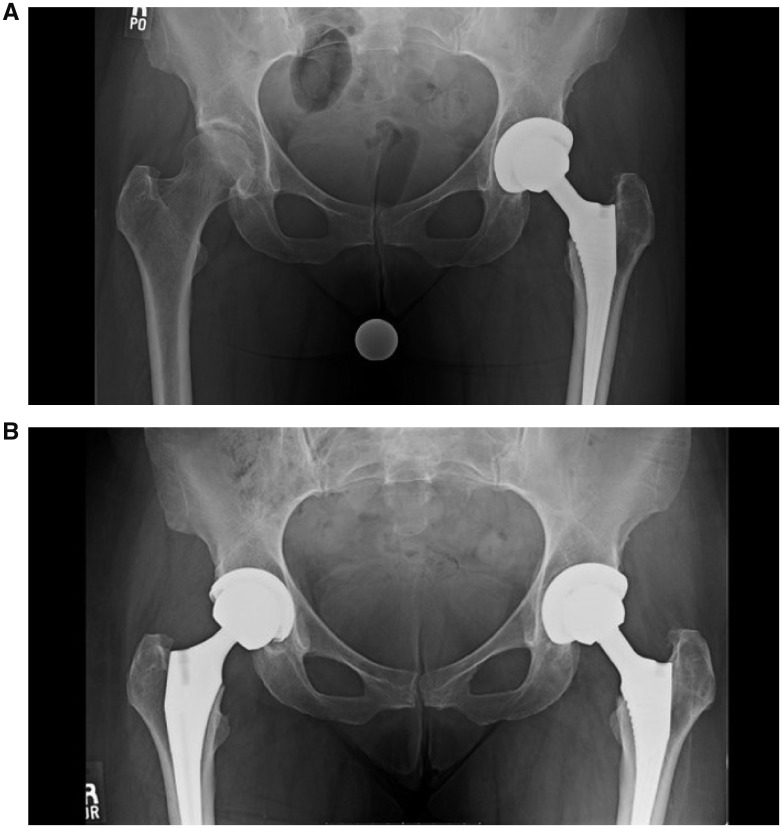
Pre-operative (**A**) and post-operative (**B**) radiographs for patients undergoing THA in the study group.

The study group demonstrated no revision arthroscopies while the control group had 4 revision arthroscopies (14.8%) performed at an average of 15.4 months post-operative (range 8.7–21.5 months), which was not statistically significant. The revisions in the control group were done for the following reasons: (i) labral re-tear; (ii) loose body, labral tear, cam lesion with alpha angle >50°, stiffness; (iii) labral re-tear; (iv) labral re-tear, hip flexor tendinitis. However, the study group was noted to have six patients converted to THA (67%) at an average of 35.8 months post-operative (SD 24.5 months), compared with only four patients (15%) in the control group, which was found to be statistically significant (*P* = 0.006). These six patients were noted to have high grade chondral injury (Grade III or IV outerbridge) at the time of their arthroscopy (four acetabular, two femoral head; see Table V). The six patients converted to THA were converted for significant radiographic progression of osteoarthritis with worsening hip symptoms (Figs 1 and 2).

**Table V.** Secondary operations performed in each group (arthroplasty and conversion to THA)

	Control (*n* = 27)	Contralateral THA (*n* = 9)	*P*-value
Revision arthroscopies (*n*, %)	4 (15%)	0 (0%)	0.553
Time to revision (months, mean, SD, range)	15.4±6.6 (8.7–21.5)	–	
Total hip arthroplasty (*n*, %)	4 (15%)	6 (67%)	**0.006**
Time to THA (months, mean, SD, range)	35.8±24.5	20.3±9.7	0.191

Bold denotes statistically significant difference.

Patient satisfaction scores with the contralateral THA in the study group did not statistically differ when comparing those that converted to THA and those that did not (8.75 versus 9.333, *P-*value = 0.72). 

## DISCUSSION

This study evaluated the outcome of hip arthroscopy for labral pathology in patients with a contralateral THA using a matched cohort of patients who had not previously had contralateral THA. Patients were matched to minimize possible confounding effects of BMI, age, gender, LCEA, Tonnis grade, Outerbridge classification, labral tear type and procedure performed. The groups demonstrated no significant differences in PROs including mHHS, NAHS, HOS-SSS, VAS and patient satisfaction scores, however, secondary to dropout due to conversion to THA, our study was not powered to detect this difference and the findings of PROs are of minimal clinical value. The study group was found to have a statistically higher conversion to THA (6/9, 67%), compared with the matched cohort (4/27, 15%). This study strongly suggests that the outcomes of primary hip arthroscopy are not as predictable in patients who had contralateral THA compared with patients with similar pathology, demographics and intraoperative findings that have not had a contralateral THA.

As noted previously, conversion to THA following arthroscopy has been reported between 4 and 25%, with patients who are older with higher rates (18.1% over 40, 23% over 50, and 25.2% for patients over 60 years old) [24–26]. This is significantly different when compared with our study group who averaged 49 years of age at the time of surgery, however, had a 67% conversion to THA. This can partially be attributed to the high level of satisfaction that the patients experienced after undergoing contralateral THA (average satisfactio*n* = 8.75 out of 10). Due to this successful endpoint, these patients are aware of the potential pain relief and functional improvement that can be gained following THA, giving them a different vantage point compared with their counterparts who have not experienced THA. Also, with THA the amount of disability in the arthroplasty hip prior to arthroplasty is predictably greater than that in patients with labral tear, so the change in function, pain and mobility is a larger change, which can lead to greater patient satisfaction [36]. Along with this, the post-operative rehabilitation is quite different between hip arthroscopy for labral pathology and THA. While after arthroscopy the patient is protected weight-bearing for a period of time with extensive physical therapy, following THA the patient is immediately weight-bearing as tolerated with typically minimal post-operative physical therapy.

Previous studies have noted that over 80% of patients indicate they are satisfied following THA [37, 38]. Studies have demonstrated similar patient satisfaction following primary hip arthroscopy [21, 22, 39, 40]. However, it is important to note that improvement for patients, in terms of pain and function, is partially affected by the level of pre-operative disability [36]. Patients who undergo THA typically have a higher level of pre-operative disability compared with those undergoing hip arthroscopy. So, while the procedures lead to improvement in both sets of patients, the magnitude of this change can be drastically different. This could partially account for the higher conversion rate to THA in the study group.

The patients who converted to THA in the study group were all noted to have outerbridge III or IV changes at the time of initial arthroscopy. Pre-operative imaging did not demonstrate this extent of articular cartilage damage (radiographs or MR arthrogram). A previous study by Bragdon et al. demonstrates poor results following hip arthroscopy for labral tears patients with increased age or higher outerbridge classification at the time of surgery [41]. This makes diagnosis of cartilage damage crucial to operative selection of patients undergoing hip arthroscopy. MR arthrography has been found to have a sensitivity and specificity of 77 and 79%, respectively, for diagnosis of femoral head and acetabular cartilage lesions [42]. This demonstrates the difficulty of diagnosis of these lesions prior to hip arthroscopy.

To the author’s knowledge, there is no literature published to date assessing success of hip arthroscopy in patients with contralateral total hips. The strength of this study is the matched-pair analysis limiting confounding variables such as age, sex, gender, BMI, LCEA, Outerbridge classification and procedure type. Age and level of degenerative changes have been found to have a significant influence on the results of hip arthroscopy [20]. The weaknesses of the paper include the weaknesses of a retrospective chart review. Due to the extreme rarity of this case, as demonstrated by it accounting for only 0.67% of cases in these surgeons practice and likely in general throughout hip arthroscopy, low numbers of patients are available for analysis. Another weakness of this study is the lack of power to detect a difference in PROs, largely secondary to patient dropout (conversion to THA). However, the major strength of this study is that it is the first study in the literature to identify the poor results (high conversion to THA) in patients who are treated with hip arthroscopy following contralateral THA. 

In conclusion, arthroscopy cannot be currently recommended in patients who have undergone contralateral THA due to the high conversion to THA (67%).
